# Supplementary material for: Hypoxia Differently Affects TGF-β2-Induced Epithelial Mesenchymal Transitions in the 2D and 3D Culture of the Human Retinal Pigment Epithelium Cells
Source: Int J Mol Sci. 2022 May 13;23(10):5473. doi: 10.3390/ijms23105473 (PMC9143417; doi:10.3390/ijms23105473)
Supplement: Supplementary file 1 [file ijms-23-05473-s001.zip › ijms-1686693-supplementary.pdf]

Supplemental Table S1. The sequences of primers and probes used in this study.

		Sequence	Exon Location	RefSeq Number
human RPLP0[*1]	Probe	5'-/56-FAM/CCCTGTCTT/ZEN/CCCTGGGCATCAC/3IABkFQ/-3'	2-3	NM_001002
	Primer2	5'-TCGTCTTTAAACCCTGCGTG-3'		
	Primer1	5'-TGTCTGCTCCCACAATGAAAC-3'		
human COL1A1[*1]	Probe	5'-/56-FAM/TCGAGGGCC/ZEN/AAGACGAAGACATC/3IABkFQ/-3'	1-2	NM_000088
	Primer2	5'-GACATGTTCAGCTTTGTGGAC-3'		
	Primer1	5'-TTCTGTACGCAGGTGATTGG-3'		
human COL4A1[*1]	Probe	5′-/56-FAM/TCATACAGA/ZEN/CTTGGCAGCGGCT/3IABkFQ/-3′	51-52	NM_001845
	Primer2	5′-AGAGAGGAGCGAGATGTTCA-3′		
	Primer1	5′-TGAGTCAGGCTTCATTATGTTCT-3′		
human COL6A1[*1]	Probe	5′-/56-FAM/CAGGTTTCG/ZEN/GTCACAGCGGTAGT/3IABkFQ/-3′	2-3	NM_001848
	Primer2	5'-CCTCGTGGACAAAGTCAAGT-3'		
	Primer1	5'-GTGAGGCCTTGGATGATCTC-3'		
human FN1[*1]	Probe	5′-/56-FAM/TACAGCTTA/ZEN/TTCTCCCTCGCCCAG/3IABkFQ/-3′	3-4	NM_212482
	Primer2	5'-CGTCCTAAAGACTCCATGATCTG-3'		
	Primer1	5'-ACCAATCTTGTAGGACTGACC-3'		
human HIF1A[*2]	Primer2	5′-CTCTGATCATCTGACCAAAACTCA-3′	8-9	NM_181054
	Primer1	5′-CAACCCAGACATATCCACCTC-3′		
human HIF2A[*2]	Primer2	5'-AGCCTATGAATTCTACCATGCG-3'	7-8	NM_001430
	Primer1	5'-CTTTGCGAGCATCCGGTA-3'		
human PGC1A[*1]	Probe	5′-/56-FAM/ACCAGCCTC/ZEN/TTTGCCCAGATCTTC/3IABkFQ/-3′		NM_003299
	Primer2	5'-TGTCTGTATCCAAGTCGTTCAC-3'		
	Primer1	5'-GAGTCTGTTATGGAGTGACATCG-3'		
human STAT3[*1]	Probe	5′-/56-FAM/AGCTGCACC/ZEN/TGATCACCTTTGAGAC/3IABkFQ/-3′	14-16	NM_213662
	Primer2	5'-AGGCATTTGGCATCTGACAG-3'		
	Primer1	5'-TGCTTCCCTGATTGTGACTG-3'		
human IL6[*1]	Probe	5′-/56-FAM/CAACCACAA/ZEN/ATGCCAGCCTGCT/3IABkFQ/-3′	4-5	NM_000600
	Primer2	5'-GCAGATGAGTACAAAAGTCCTGA-3'		
	Primer1	5'-TTCTGTGCCTGCAGCTTC-3'		
Human FOS[*1]	Probe	5′-/56-FAM/CAGCCGACT/ZEN/CCTTCTCCAGCATG/3IABkFQ/-3′	1-2	NM_010234
	Primer2	5'-GGCACTAGAGACGGACAGAT-3'		
	Primer1	5'-ACAGCCTTTCCTACTACCATTC-3'		
Human TGF β 1[*1]	Probe	5′-/56-FAM/ACCCGCGTG/ZEN/CTAATGGTGGAA/3IABkFQ/-3′	1-2	NM_000660
	Primer2	5′-GTTCAGGTACCGCTTCTCG-3′		
	Primer1	5′-CCGACTACTACGCCAAGGA-3′		
Human AGT[*1]	Probe	/56-FAM/AGAAACCTC/ZEN/TCATCGTTCCTTGGAAGTG/3IABkFQ/	2-3	NM_007428
	Primer2	CACAGACACCGAGATGCT		
	Primer1	GCACCCTACTTTTCAACACCTA		
Human MYC[*1]	Probe	/56-FAM/CGGTGTCTC/ZEN/CTCATGCAGCACT/3IABkFQ/	2-3	NM_013261
	Primer2	CTTCCCTCATCTTCTTGCTCTTCT		
	Primer1	TTCTCTCCTTCCTCGGACTC		

*1 Taqman probes (IDT, Coralville, IA, USA). *2 SYBR probes (IDT, Coralville, IA, USA).
